# Comparison of Medical and Consumer Wireless EEG Systems for Use in Clinical Trials

**DOI:** 10.3389/fnhum.2017.00398

**Published:** 2017-08-03

**Authors:** Elena Ratti, Shani Waninger, Chris Berka, Giulio Ruffini, Ajay Verma

**Affiliations:** ^1^Biogen Cambridge, MA, United States; ^2^Advanced Brain Monitoring, Inc. Carlsbad, CA, United States; ^3^Neuroelectrics Corporation Cambridge, MA, United States

**Keywords:** neurophysiology, electrophysiology, electroencephalogram, quantitative EEG, clinical trials, consumer EEG

## Abstract

**Objectives:** To compare quantitative EEG signal and test-retest reliability of medical grade and consumer EEG systems.

**Methods:** Resting state EEG was acquired by two medical grade (B-Alert, Enobio) and two consumer (Muse, Mindwave) EEG systems in five healthy subjects during two study visits. EEG patterns, power spectral densities (PSDs) and test/retest reliability in eyes closed and eyes open conditions were compared across the four systems, focusing on Fp1, the only common electrode. Fp1 PSDs were obtained using Welch's modified periodogram method and averaged for the five subjects for each visit. The test/retest results were calculated as a ratio of Visit 1/Visit 2 Fp1 channel PSD at each 1 s epoch.

**Results:** B-Alert, Enobio, and Mindwave Fp1 power spectra were similar. Muse showed a broadband increase in power spectra and the highest relative variation across test-retest acquisitions. Consumer systems were more prone to artifact due to eye blinks and muscle movement in the frontal region.

**Conclusions:** EEG data can be successfully collected from all four systems tested. Although there was slightly more time required for application, medical systems offer clear advantages in data quality, reliability, and depth of analysis over the consumer systems.

**Significance:** This evaluation provides evidence for informed selection of EEG systemsappropriate for clinical trials.

## Introduction

Neurodegenerative disorders represent huge global unmet medical needs and require the development of new disease modifying therapies. Given the insidious nature of these disorders and the high cost of many diagnostic tests, there is a significant need for widely available, reliable, and inexpensive biomarkers to track progression of neurodegenerative processes in time frames suitable for drug development. In this context, EEG may have remarkable potential. Although EEG is susceptible to known lifestyle factors and medications, it has many considerable advantages. EEG reflects synaptic activity, which is a common denominator for the functional impact of neurodegenerative processes. EEG is a non-invasive, portable, safe, and inexpensive technology that is widely accepted and requires relatively short acquisition time. Qualitative EEG is routinely utilized in clinical practice for the diagnosis of epilepsy. More recently, an integration of a quantitative EEG biomarker (qEEG) and clinician's evaluation have been proposed for the assessment of attention-deficit/hyperactivity disorder (ADHD) and has been granted from the FDA (Food and Drug Administration) class II designation to support the clinical evaluation of ADHD (Lenartowicz and Loo, 2014; Snyder et al., 2015). qEEG is in investigational stages for use as an endpoint in neurodegenerative diseases in clinical trials. However, recent advances in data analyses, interpretation and improved spatial resolution have increased the potential of EEG as a reliable, accurate biomarker for neurodegenerative disease progression. Many reported observational resting state qEEG analyses support its potential value as a biomarker for detection of neural signatures of neurodegeneration occurring in Alzheimer's disease (Babiloni et al., 2011; Moretti et al., 2011; Berka et al., 2014; Chen et al., 2015; Garn et al., 2015; Ruffini et al., 2016; Waninger et al., 2016), Parkinson's disease (Sarnthein and Jeanmonod, 2007; Babiloni et al., 2011; Soria-Frisch et al., 2014; Shani Waninger et al., 2015; Kroupi et al., 2017) and frontotemporal dementia (Pijnenburg et al., 2008; Nishida et al., 2011; Caso et al., 2012; Goossens et al., 2016).

Over the years, EEG hardware technology has also evolved and several wireless multi-channel systems have emerged that deliver high quality EEG and physiological signals in a simpler, more convenient and comfortable design than the traditional, cumbersome systems. Traditional EEG systems require lengthy assembly and application time, typically involving abrasion of the patient's scalp. The application time and discomfort render these traditional systems challenging to use in populations affected by dementia, where cooperation with lengthy clinical procedures is often difficult. However, several currently available wireless systems can be applied in 20 min or less with no discomfort during application and with a comfortable fit during acquisitions. Combined with advances in signal detection and quantitative analysis techniques, wireless systems are ideal candidates for relatively rapid, tolerable clinical assessment of potentially challenging dementia populations, such as behavioral variant frontotemporal dementia, characterized by prominent behavioral and personality changes.

More recently, there has also been a growing market for consumer wearable technologies leading to limited-channel systems available for personal use, such as meditation and relaxation training. It is conceivable that these systems, albeit their limited coverage, may also be used in selected clinical studies. Application of these consumer systems in clinical trials research has not been extensively explored however and the accuracy and reliability of these systems for repeated measurements have not been well-established. Further, it is not clear whether the limited-channel acquisition may provide sufficient data and anatomical coverage to assess the neural signatures in patients affected by neurodegenerative diseases.

The current study was designed to provide initial evaluation of the potential of consumer EEG systems for clinical trials, by comparing the ease of use, accuracy and reliability of two medical grade, multi-channel wireless EEG systems, B-Alert X24, and Enobio 20, with two consumer, limited-channel systems, Muse and Mindwave.

## Materials and methods

### Participants

The study population included five healthy participants who met eligibility for the study (Table 1). Subjects were excluded if, after review of their medical history, concomitant medications, and lifestyle (alcohol and caffeine consumption as well as smoking status), they were not considered healthy. Specifically, they were excluded from the study if they had a history of epilepsy, or other sleep, neurological or psychiatric disorders or were taking medications or had a lifestyle known to affect EEG signal such as smoking and considerable alcohol and caffeine consumption.

**Table 1:** Study population demographics and baseline characteristics.

	**Subject 1**	**Subject 2**	**Subject 3**	**Subject 4**	**Subject 5**	**Group measures^a^**
Age (years)	25	23	27	37	23	27 ± 5.8
Gender (male)	Male	Male	Female	Male	Female	60%
Handedness (right)	Right	Right	Right	Right	Right	100%
Education (years)	17 Undergraduate Degree	17 Undergraduate degree	17 Undergraduate degree	19 Advanced Degree	17 Undergraduate degree	17.4 ± 0.9
Caffeine consumption (number of coffees/day)	2–3	1–2	1	1–2	0–1	1.8 ± 0.8
Alcohol consumption (number of drinks/day)	0	0	1	0–2	0–1	0.8 ± 0.8
Smoking	No	No	No	No	No	0%

a *Categorical variables: N (%); Continuous variables: Mean ± Standard Deviation (SD)*.

Written informed consent was obtained from all the study participants following the guidelines for experimental investigation with human subjects required by the Chesapeake Institutional Review Board.

### Study procedures, EEG data acquisition, and analysis

To avoid potential EEG signal variability associated with nutritional intake and circadian variations, all visits occurred in the morning following a standard low carbohydrate, high protein breakfast (to minimize post-prandial drowsiness) at the Advanced Brain Monitoring (ABM) research labs study site. In addition, participants were asked to avoid alcoholic beverages the night before the study visit as well as to fast and avoid caffeine on the morning of the visit.

**Figure 1:**
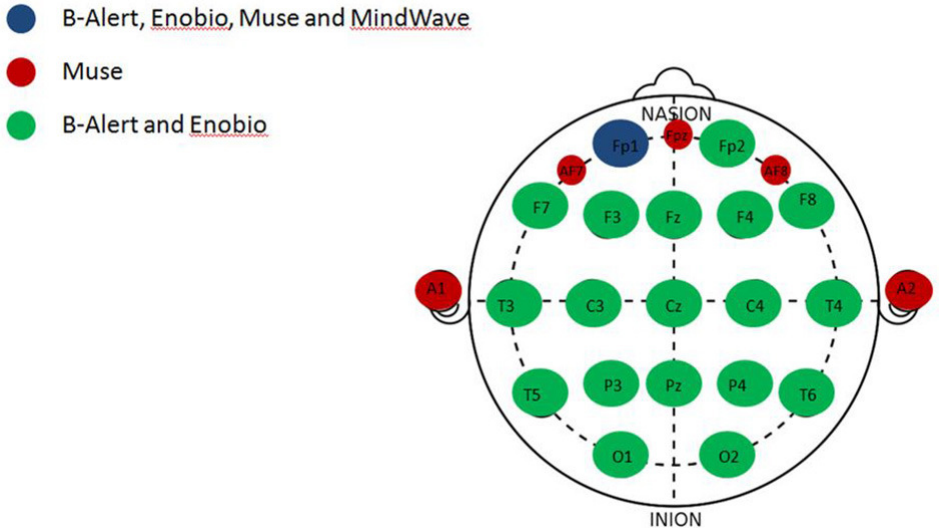
Anatomical channel distribution of the multi- and limited-channel EEG systems. Representation of the shared and unique channels among the four EEG systems evaluated. The common channel to all the four EEG systems, Fp1 (blue); channels unique to the Muse system (red); channels shared between the multi-channel systems, B-Alert and Enobio (green).

EEG data was acquired during two separate visits ~1 week apart from two multi-channel (20 channels) medical EEG systems, B-Alert (Advanced Brain Monitoring, ABM) and Enobio (Neuroelectrics) (Figure 1, Table 2) and two limited-channel consumer systems, Muse (Interaxon, 2 channels) and Mindwave (Neurosky, one channel) (Figure 1, Table 2) in the following order: Muse, Mindwave, B-Alert, Enobio. The B-Alert X24 EEG System and Enobio are both CE medically certified 20 channel wireless systems applied in the standard international 10–20 montage and acquire EEG signal at a sampling rate of 256 Hz. In addition, B-Alert has been cleared by the FDA for use as a medical device.

**Table 2:** EEG systems comparisons.

	**Muse**	**Mindwave**	**B-Alert X24**	**Enobio 20**
	** 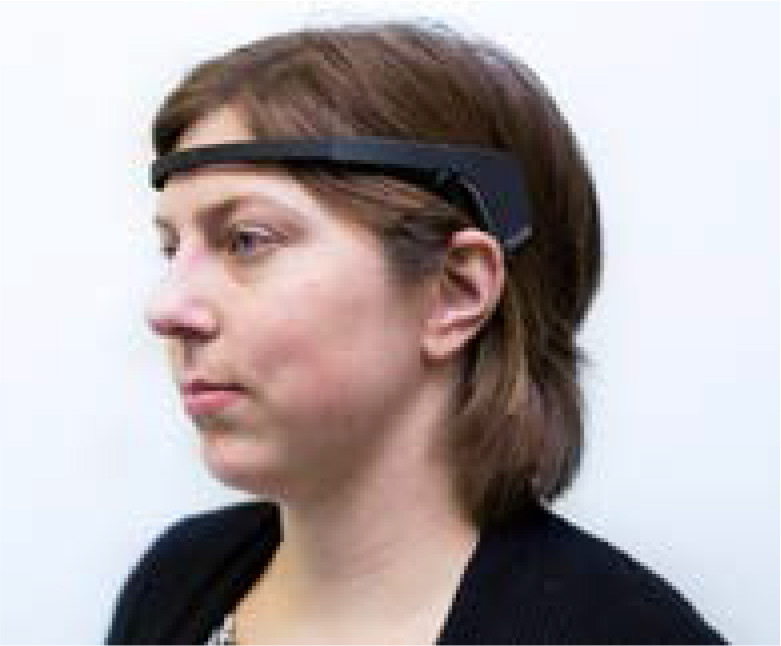 **	** 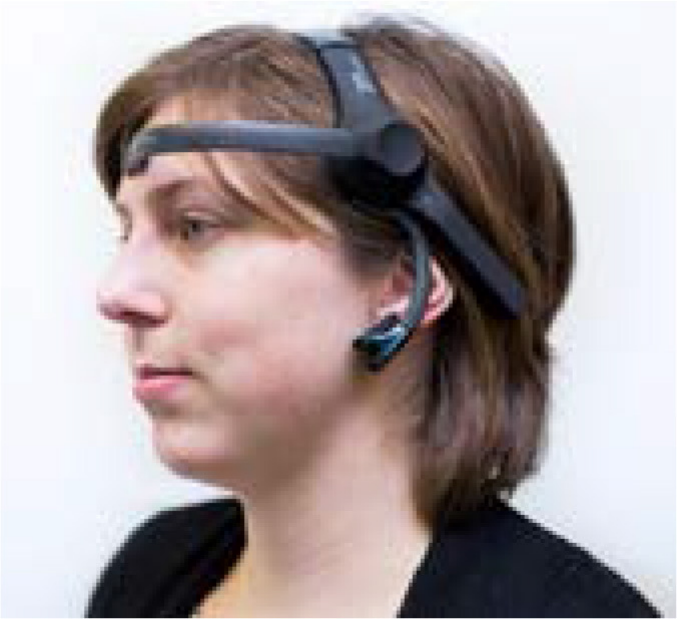 **	** 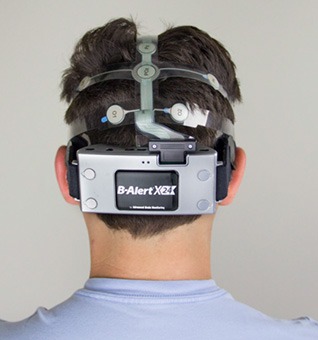 **	** 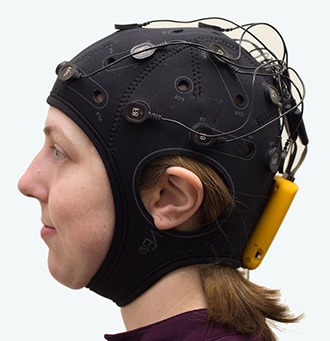 **
**Company**	**Interaxon**	**NeuroSky**	**Advanced brain monitoring**	**Neuroelectrics**
Channels	2	1	20	20
Sampling rate	220 Hz	512 Hz	256 Hz	500 Hz
Wet/dry electrodes	Dry	Dry	Wet	Wet
Signal quality check	Yes	Yes	Yes	Yes
Impedance check	n/a	n/a	Yes	Yes
Setup time (minutes)	5	3	20–25	20–25

B-Alert uses mastoids as a reference channel. Enobio can be used with a mastoid reference, ear-clip or using other scalp locations. Mindwave also has a reference on the ear slip and Muse has three reference channels on the forehead. All the systems included signal quality check, however as opposed to the medical EEG systems, both consumer systems have dry electrodes and no impedance check.

These technologies were selected based on their ability to provide end-users with raw EEG outputs through practical and non-cost-prohibitive access.

Ten minutes of resting state EEG was acquired during eyes open (EO) with visual fixation on a cross symbol presented on a computer screen (5 min) as well as while eyes closed (EC) for 5 min.

Since all four systems only share the FP1 channel (Figure 1), EEG patterns (raw and decontaminated), power spectral densities (PSDs), and test/retest reliability comparisons across the four EEG systems evaluated in the study were performed focusing on the Fp1 electrode in both EO and EC conditions.

The EEG data from each system were loaded into MATLAB (Mathworks) using custom built functions. The EEG data recorded during breaks and instructions were discarded prior to analysis. Power spectral density of EEG for each 1 s epoch was calculated using Welch's modified periodogram method with a Hamming window tapering of 1 s length. PSDs were calculated on resting EEG during both eyes open and eyes closed periods. To allow equal units (μV), a correction factor of 1.25 (Muse), 0.25 (MindWave), or 1,000 (Enobio 20) was applied. All Fp1 channel PSD data were averaged for the five subjects for each visit. Test-retest was performed by calculating and plotting Vist1/Visit2.

## Results

### Participants

Participants were all healthy volunteers with an average age of 27 years, they were all right handed and non-smokers, and predominantly males (60%). Their demographics and baseline characteristics are summarized in Table 1.

### Resting state EEG

EEG data was successfully collected from all four systems tested. As expected, due to the dry electrodes and the limited number of channels, the set up time was considerably less for the consumer EEG systems. However, there was no observed difference in subjects' tolerance and acceptance across the four systems.

**Figure 2:**
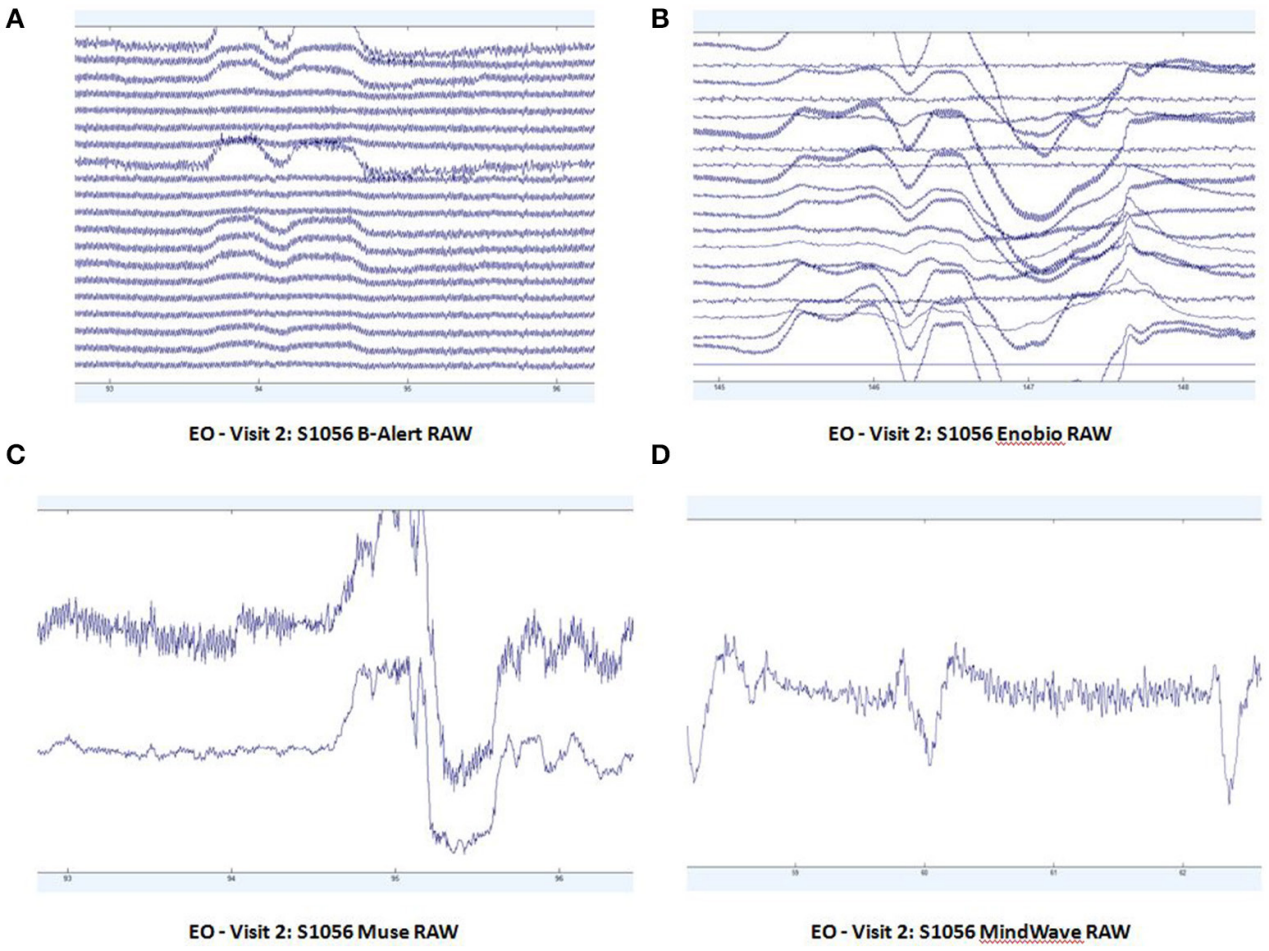
Extracts of EO raw data from B-Alert **(A)** and Enobio **(B)** Muse **(C)** and MindWave **(D)** within the same participant during the same visit.

**Figure 3:**
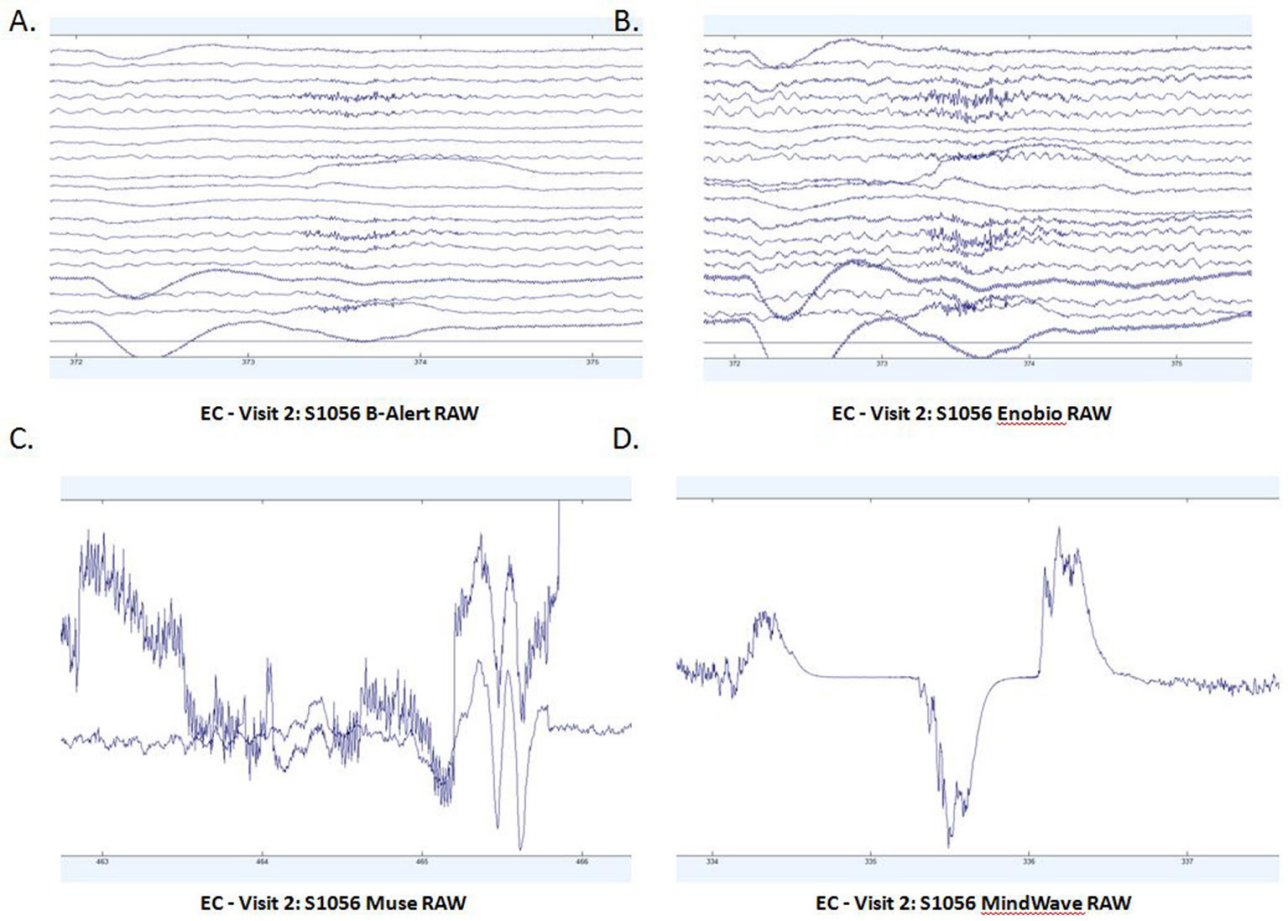
Extracts of EC raw data from B-Alert **(A)** and Enobio **(B)** Muse **(C)** and MindWave **(D)** within the same participant during the same visit.

Patterns of raw EEG data in EO (Figure 2) and EC (Figure 3) conditions were collected and evaluated from each system. Muse and Mindwave were more prone to artifact due to eye blinks and muscle movement in the frontal region with eye opening (Figure 2).

### Fp1 channel power spectral densities (PSDs) comparison

**Figure 4:**
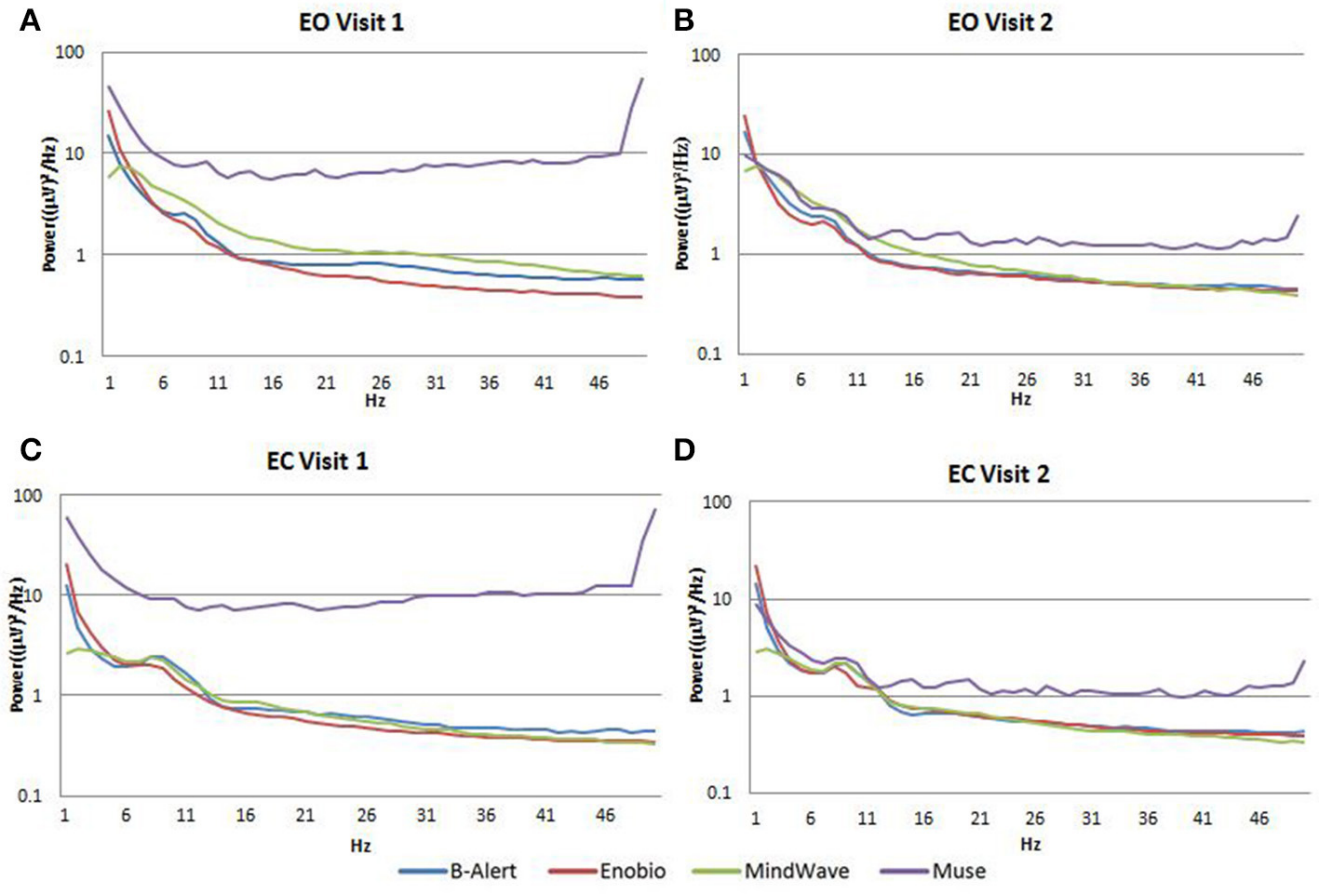
Fp1 Power Spectral Densities (PSDs) from all four EEG systems in the EO condition at Visit 1 **(A)** and Visit 2 **(B)** and EC condition at Visit 1 **(C)** and Visit 2 **(D)**.

Average PSDs in the Fp1 channel common to all the four EEG systems for Visit 1 and Visit 2 were calculated and were plotted for both EO (Figures 4A,B) and EC (Figures 4C,D) conditions. In the EO condition, B-alert and Enobio spectra were approximately equal, while Mindwave was slightly increased but followed a similar curve. A broadband increase in power was observed for PSDs acquired with the Muse system. Similarly, in the EC condition (Figures 4C,D), B-Alert, Enobio, and Mindwave PSDs were similar however increased broadband power was observed for Muse spectra, which also appeared to have higher variation than the other systems. Peaks at 8–12 Hz (alpha band) were visible in the spectra acquired with the B-Alert, Enobio, and Mindwave systems at each visit. While there was a clear alpha peak for the Muse power spectra on Visit 2, no peak was observed in the Visit 1 spectra.

### Fp1 channel resting EEG test/retest comparison

**Figure 5:**
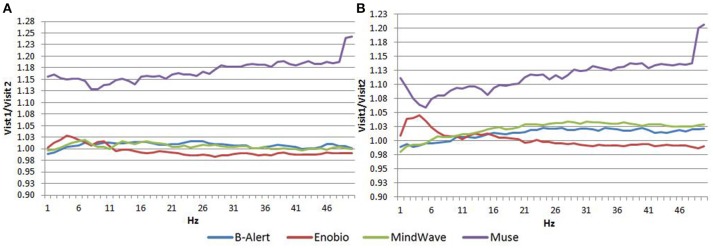
Test-retest ratios for EC **(A)** and EO **(B)** condition.

In the EC condition, the power spectral ratio was between 0.975 and 1.025 for B-Alert, Enobio, and Mindwave (Figure 5A). The Muse system PSD ratios had more variation then the other three systems with ratios between 1.125 and 1.225. In the EO condition, there appeared to be slightly more variation for Enobio in the slow waves Delta (1–3 Hz), Theta (3–7 Hz), and slow alpha (8–10) and for B-Alert and Mindwave in the faster waves Beta (13–30 Hz) and Gamma (25–40 Hz). However, ratios were still between 0.975 and 1.05. Similarly to the EC condition, the test/retest for Muse had higher variation, with ratios up to 1.2 (Figure 5B).

## Discussion

This study compared quantitative EEG signal and test-retest reliability of medical and consumer EEG systems in order to evaluate their potential application in clinical research and clinical trials. Newly popularized consumer EEG systems were evaluated due to their low cost, wide accessibility, and potential for home based studies in challenging populations. Among the popular applications of consumer EEG systems are meditation and relaxation training as well as coping with anxiety or pain. Recent investigations have been exploring their utility beyond gaming: NeuroSky was shown to be able to detect onset of stage 1 sleep (Van Hal et al., 2014) and there has also been interest in assessing consumer EEG within brain computer-interfaces (Bialas and Milanowski, 2014; Kim et al., 2015; Taherian et al., 2017). The application of simpler EEG systems (6-channels, for example) is also being explored for emergency settings (Jakab et al., 2014). A prior evaluation, although utilizing a more complex headset, showed that commercially available multi-lead consumer EEG systems, such as the Emotiv EPOC 16-electrode cap, may also have value in evaluating clinical conditions (Schiff et al., 2016).

This study provided evidence that fairly good quality EEG data can be successfully collected from consumer EEGs. However, there were distinctions in the power increase, test retest, and shape of the alpha peak observed at 8–13 Hz. Mindwave showed overall similar Fp1 power spectra to the medical systems with a slight broadband increase over B-Alert and Enobio. Muse showed a broadband increase in power spectra, which may reflect artifact in the data acquired by a dry electrode.

Consumer EEG systems did show a significantly more convenient and faster set up,which is optimal for their intended use in entertainment and self-help applications. However, their data quality was overall negatively affected by artifact susceptibility associated with the dry electrode. As expected, the data quality was particularly diminished during EO. The lack of impedance testing capability and application to the frontal region, which is particularly prone to eye blinks and muscle movement with eye opening also likely contributed to this relative artifact. Additionally, the assessment performed by consumer EEG systems is, by their nature, limited and confined to the only anatomical brain region covered by the few channels, precluding multi-networks evaluations.

Dry electrodes may also be more prone to result in discomfort over time and pose a higher risk of misplacement on the forehead, leading to inaccurate signal acquisition and test/retest. Compared to medical grade equipment, test/retest reliability was lower in consumer EEG systems. Reliability was measured with a test-retest acquisition for all systems (Figure 5). While B-Alert, Enobio, and Mindwave performed reasonably well, Muse had relatively low reliability. Further, while a clear alpha peak was demonstrated for Muse on Visit 2 (Figure 4), the absence of an alpha peak on Visit 1 suggests a lack of consistency that may be due to artifact. Consistent reliable measures of brain activity are crucial in clinical trials when monitoring disease progression and evaluating efficacy of an experimental therapeutic. While the consumer systems may be useful for a quick assessment when time is limited, these limitations of consumer EEG could hinder their applications in research and clinical trials settings and a medical grade system with high test-retest reliability is recommended for use as a pharmacodynamic endpoint in clinical assessments.

Ultimately, the comparison of medical and consumer EEG systems under experimental conditions highlighted differentiation in performance and, particularly, specific limitations of use that could hinder the applications of consumer systems in research and clinical trials settings. On the other hand, the medical multi-lead systems are less ideal for entertainment purposes that require rapid setup and data processing from a minimal number of electrodes. The main limitation of this study relies in the small size of the study population and lack of randomization of order of systems used for acquisition. Data was acquired in the same order of systems in order to avoid effects of time of acquisition that can impact EEG due to circadian rhythms. However, EEG power metrics have proven to be a very reliable and repeatable for individuals when using standard EEG systems. For this reason, the selected sample size was considered informative.

In conclusion, EEG data can be successfully collected from all four systems tested, including consumer EEG systems, with varying limitations on usability, data quality and reliability that guide their optimal applications including in clinical trial settings. Susceptibility to artifact and variability in test/retest reliability associated with current consumer EEG systems suggest the use of medical grade EEG system for robust clinical cross-sectional and longitudinal EEG data collections.

## Author contributions

ER and AV: participated in the conception and the design of the project, the analysis, and interpretation of data and manuscript preparation. SW, CB, and GR: participated in the conception and the design of the project, its execution, the analysis and interpretation of data and manuscript preparation. All authors have been involved in drafting, writing, and revising the manuscript and they all have read and approved the final version of the manuscript.

### Conflict of interest statement

ER and AV: Biogen employee. SW and CB: Advanced Brain Monitoring, Inc employee. GR: Neuroelectrics employee.
